# Konsensus-Statement der Österreichischen Gesellschaft für Schlafmedizin (ÖGSM/ASRA) zum Management der residualen exzessiven Tagesschläfrigkeit (rETS) bei obstruktiver Schlafapnoe: Die SPAIN-Checkliste für den klinischen Alltag

**DOI:** 10.1007/s11818-022-00359-3

**Published:** 2022-07-09

**Authors:** O. Amouzadeh-Ghadikolai, R. Popovic, A. Heidbreder, A. Kugi, M. Saletu

**Affiliations:** 1 Abteilung für Psychiatrie und Psychotherapie 1, LKH Graz II – Standort Süd, Graz, Österreich; 2 Abteilung für Innere Medizin, Franziskusspital, 1050 Wien, Österreich; 3 Universitätsklinik für Neurologie, Medizinische Universität Innsbruck, Anichstraße 35, 6020 Innsbruck, Österreich; 4 Abteilung für Innere Medizin, LKH Villach, 9500 Villach, Österreich; 5 Bereich Schlafmedizin, Abteilung für Neurologie, LKH Graz II – Standort Süd, Graz, Österreich

**Keywords:** Obstruktive Schlafapnoe, Residuale exzessive Tagesschläfrigkeit, ETS, CPAP, Narkolepsie, Obstructive sleep apnea, Residual excessive daytime sleepiness, EDS, CPAP, Narcolepsy

## Abstract

**Hintergrund:**

Sechs bis zehn Prozent aller Patienten mit einer obstruktiven Schlafapnoe leiden trotz adäquater nächtlicher Überdrucktherapie oder einer effektiven Alternativtherapie an einer residualen exzessiven Tagesschläfrigkeit (rETS). Die Differentialdiagnose der rETS stellt im klinischen Alltag eine interdisziplinäre Herausforderung dar.

**Fragestellung:**

Entwicklung eines übersichtlichen Leitfadens für die Erkennung, die differentialdiagnostischen Abwägungen und die Möglichkeiten der weiterführenden Behandlung der rETS in der klinischen Praxis.

**Material und Methode:**

MeSH-Analyse basierte Recherche und interdisziplinäre Abstimmung von Fachvertreter:innen der Inneren Medizin und Pneumologie, Neurologie sowie Psychiatrie und Psychotherapie.

**Ergebnisse:**

SPAIN-Checkliste zur systematischen differentialdiagnostischen Exploration der rETS mit den Parametern: *S* chlafverhalten, *P* sychische Ursachen, *A* namnese der Medikation, *I* nternistische Ursachen und *N* eurologische Ursachen.

**Schlussfolgerungen:**

Die rETS sollte als behandlungswürdiges Symptom erkannt werden. Sie verlangt nach einer interdisziplinären Abklärung und der individuellen Abstimmung der Behandlung auf die Bedürfnisse der Betroffenen.

## Abstract (en)

**Background:**

Six to ten percent of patients with obstructive sleep apnea suffer from residual excessive daytime sleepiness (rEDS) despite adequate nocturnal positive airway pressure therapy or effective alternative treatment. The differential diagnosis of rEDS is an interdisciplinary challenge in clinical practice.

**Objectives:**

Development of a clear guideline for the detection, differential diagnostic considerations, and options for the continuing treatment of rEDS in clinical practice.

**Methods:**

MeSH analysis-based research and interdisciplinary consensus among specialists in internal medicine and pneumology, neurology, as well as psychiatry and psychotherapy.

**Results:**

The SPAIN checklist for systematic differential diagnostic exploration of rEDS with the parameters: *S* leep behavior, *P* sychological causes, *A* namesis of medication, *I* nternal causes and *N* eurological causes.

**Conclusions:**

rEDS should be recognized as a symptom worthy of treatment. It requires an interdisciplinary assessment and the individual adaptation of the treatment to the needs of the affected person.

Bei 6–10 % aller Patienten mit einer obstruktiven Schlafapnoe tritt trotz adäquater nächtlicher Überdrucktherapie oder einer effektiven Alternativtherapie eine residuale exzessive Tagesschläfrigkeit (rETS) auf. Die differentialdiagnostische Abklärung und Behandlungsmöglichkeiten der rETS stellen selbst den erfahrenen Schlafmediziner vor eine Herausforderung. Um ein adäquates Management der rETS in Schlafzentren zu gewährleisten, wurde von der rETS-Task Force der Österreichischen Gesellschaft für Schlafmedizin (ÖGSM/ASRA) der vorliegende alltagsorientierte Leitfaden erarbeitet und in einer Checkliste (SPAIN-Checkliste) zusammengefasst.

## Einleitung

Etwa 30 % der österreichischen Bevölkerung leiden an Tagesschläfrigkeit, wobei die obstruktive Schlafapnoe (OSA) zu den häufigsten Ursachen zählt [46]. In den meisten Fällen kommt es durch eine nächtliche Überdrucktherapie (engl. continuous positive airway pressure [CPAP]) zu einer Verbesserung der Tagesschläfrigkeit. Gegenwärtig geht man jedoch davon aus, dass 6–10 % der OSA-Patienten trotz regelmäßiger CPAP-Therapie oder Einsatz alternativer Therapien (z. B. mit einer mandibulären Protrusionsschiene, einer HNO-chirurgischen Intervention oder einer Hypoglossusnervstimulation) weiterhin unter einer residualen Tagesschläfrigkeit (rETS) leiden [10, 29]. Kennzeichnend für eine exzessive Tagesschläfrigkeit ist ein gesteigertes Schlafbedürfnis am Tage, das zu einer erhöhten, oft nicht zu unterdrückenden Einschlafneigung bis hin zu unabwendbaren „Schlafattacken“, besonders in monotonen, zum Teil aber auch inadäquaten Situationen führen kann.

Im Behandlungsverlauf der OSA sollte eine rETS erkannt werden, da sie mit einer verminderten Lebensqualität und Leistungsfähigkeit [11, 34, 38], Minderung der CPAP-Therapieadhärenz aufgrund des nicht spürbaren Therapieeffektes [35], einem erhöhten Unfallrisiko [31] sowie möglicherweise auch einem erhöhten Risiko für kardiovaskuläre Ereignisse [24, 45] einhergeht.

Als Prädiktoren der rETS konnten bisher folgende Faktoren identifiziert werden [6, 19, 40]:

- Hoher Ausgangs-Epworth-Sleepiness-Score (ESS)
- Niedriger Apnoe Hypopnoe-Index (AHI) vor der Therapieeinleitung
- Junges Alter bei Diagnose der OSA
- Kurze Einschlaflatenz
- Unzureichende CPAP-Anwendung
- Tiefschlafdefizit
- Begleitende Herz-Kreislauf-Erkrankung
- Komorbider Diabetes mellitus
- Vermehrter Tagschlaf (bereits vor Start der CPAP-Therapie)

Ziel der rETS-Task Force der ÖGSM war es, einen für die Praxis übersichtlichen Leitfaden für die Erkennung, die differentialdiagnostischen Abwägungen und Möglichkeiten der weiterführenden Behandlung der residualen Tagesschläfrigkeit zu entwickeln. Zum Zwecke der besseren Übersicht und des Einsatzes in der Praxis wurde eine Checkliste erstellt (SPAIN-Checkliste), die die wesentlichen Faktoren zusammenfasst.

Für die umfassendere Darstellung der Prävalenz, Pathophysiologie und möglicher Auswirkungen einer rETS verweisen wir auf die entsprechende Literatur [8, 10, 22, 25, 26, 29, 33].

## Methoden

Auf Initiative der ÖGSM/ASRA wurde eine interdisziplinäre Arbeitsgruppe zur Erstellung des vorliegenden Leitfadens gebildet. Diese setzt sich aus Fachvertreter:innen der Inneren Medizin und Pneumologie, Neurologie sowie Psychiatrie und Psychotherapie zusammen. Die verwendete Literatur wurde auf Grundlage einer MeSH-Analyse basierten Recherche zusammengestellt. Aus dem pubmed-Input („Sleep Apnea, Obstructive“[Mesh] AND „residual excessive daytime sleepiness“) OR („Sleep Apnea, Obstructive“[Mesh] AND „residual excessive sleepiness“) OR („Sleep Apnea, Obstructive“[Mesh] AND „residual sleepiness“) ließen sich 55 Arbeiten finden, zu welchen weitere 24 Arbeiten nach sachbezogener Recherche hinzugesellt wurden.

Die abschließende Konsensusfindung erfolgte auf der Basis einer interdisziplinären Diskussion im Rahmen wiederholter Treffen, an welchen alle Autoren teilgenommen haben.

## Quantifizierung der rETS

Die gegenwärtig gebräuchlichste Methode, Tagesschläfrigkeit psychometrisch zu erfassen, ist der Einsatz der 8 Items umfassenden *Epworth Schläfrigkeitsskala *(*engl. Epworth sleepiness scale *[ESS]) [16]. In dieser wird das Maß der Schläfrigkeit in Alltagssituationen erfragt. Da es sich dabei um einen Fragebogen handelt, kann dieser jedoch nur ein subjektives Maß der Schläfrigkeit widerspiegeln. Ein Gesamtscore von ≥ 10 wird als pathologisch bewertet [16]. Bisherige Studien zeigen, dass Patienten mit einer rETS durchschnittlich einen ESS-Wert von 14 aufweisen [21].

Zur quantitativen Messung von Schläfrigkeit werden in Schlafzentren in erster Linie der *Multiple Schlaflatenztest *(*MSLT*) und der *Multiple Wachbleibetest/Maintenance of Wakefulness Test *(*MWT*) eingesetzt [1]. Im MSLT werden die Fähigkeit und Charakteristika des Einschlafens (z. B. Einschlaflatenz, Auftreten eines frühen REM-Schlafs bei einer Narkolepsie) bestimmt. Der MSLT ist somit ein diagnostisches Testverfahren, das die Einschlafbereitschaft misst. Im Gegensatz dazu wird im MWT, etwa im Rahmen der Beurteilung der Wirkung von stimulierenden Therapeutika, die Fähigkeit des Patienten gemessen, wach zu bleiben. Der MWT lässt keine ätiologische Zuordnung der Tagesschläfrigkeit zu. Beide Tagschlaftests werden aufgrund des personellen und zeitlichen Aufwandes nicht routinemäßig bzw. nur in spezialisierten Schlaflaboren durchgeführt.

## Definition der rETS

Die rETS lässt sich durch folgende Kriterien definieren:

Obligat:

- Pathologisch gesteigerte Tagesschläfrigkeit (ESS ≥ 10)
- OSA-Erkrankung unter polysomnographisch dokumentierter suffizienter CPAP-Therapie oder einer effektiven, adäquaten Alternativtherapie
- Adhärente Anwendung der CPAP- oder Alternativtherapie (Nutzung > 4 h pro Nacht und > 70 % der Nächte in den letzten 3 Monaten)
- Ausschluss von Differentialdiagnosen

Der Diagnose einer rETS muss somit obligat eine respiratorische Standardabklärung zur Sicherung einer effektiven Therapie der OSA vorangehen. Hierzu gehören die Sicherstellung einer ausreichenden Therapieadhärenz (z. B. durch Auslesen der CPAP-Adhärenzdaten) und der Nachweis einer suffizienten Therapie zumindest durch eine ambulante Polygraphie unter CPAP-Therapie mit einem 7‑Kanalgerät inkl. fortlaufender Druckmessung. Es sollte nach Berry und Sriram ein Apnoe-Hypopnoe-Index < 10/h als Nachweis einer suffizienten CPAP-Therapie angestrebt werden [2]. Als optimaler AHI gilt ein Wert < 5/h.

Im Falle einer Alternativtherapie sollte ebenfalls eine Therapieeffizienzkontrolle zumindest mittels Polygraphie erfolgen. Bei inkonsistenten, schwankenden oder nicht eindeutigen Befunden ist die Durchführung einer stationären Polysomnographie zur weiteren Differenzierung/Verifizierung vor allem auch zum Ausschluss anderer Ursachen der Tagesschläfrigkeit und zur möglicherweise notwendigen Therapieoptimierung (ggf. Maskenwechsel, exspiratorische Druckentlastung, Verordnung eines Warmluftbefeuchters, Adaptierung des Beatmungsmodus oder der Drücke, Optimierung des Protrusionsbehelfes u. Ä.) indiziert. Eine insuffiziente CPAP-Therapie kann durch eine REM-dominante OSA, Anwendungsfehler bei CPAP, durch „treatment emergent central sleep apnea“ (zentrale Apnoen unter CPAP) oder persistierende „respiratory effort-related arousals“ (RERAs) begründet sein, die einer rein polygraphischen Untersuchung entgehen können. Bei Letzteren handelt es sich um Flusslimitationen von mind. 10 s Dauer bei zu geringen Drücken, die zu Aufwachreaktionen (Arousals) führen. Sie werden nicht als Apnoen oder Hypopnoen klassifiziert, können aber dennoch mit einer vermehrten Tagesschläfrigkeit assoziiert sein. Naturgemäß können RERAs nur in einer PSG erfasst werden und erfordern eine Anpassung der Druckeinstellungen [26].

## Differentialdiagnose der rETS

Die Differentialdiagnose der rETS stellt im klinischen Alltag eine besondere Herausforderung dar.

Nach dem obligaten Nachweis einer suffizienten Therapie der OSA mittels respiratorischer Standarduntersuchungsverfahren (s. Definition rETS) ist eine systematische differentialdiagnostische Exploration notwendig. Diese sollte die wesentlichen Daten zum Schlafverhalten und mögliche internistische, psychiatrische und neurologische Erkrankungen, die eine Tagesschläfrigkeit erklären könnten, beinhalten. Hierbei sind grundsätzlich auch die verschiedensten fachspezifischen Leitlinien zu berücksichtigen.

Um die Exploration nach einem möglichst standardisierten Schema durchzuführen, wurde die SPAIN-Checkliste entwickelt, die folgende Parameter beinhaltet (s. Tab. 1):

**Table 1:** 

	Schlüsselfragen		Empfehlungen
**S**chlafverhalten			
Schlafmangel, BIISS Schlaf-Wach-Rhythmusstörung Schlaflosigkeit	*Wann wird das Licht abgedreht, um zu schlafen? Wann schlafen Sie ein?* *Wann wird das Licht aufgedreht, um aufzustehen? Wann wachen Sie auf? Wie oft wachen Sie auf?* *Wie viele Stunden Schlaf ergeben sich unter der Woche/am Wochenende?*	*→*	*Schlaftagebuch* *Aktigraphie*
**P**sychiatrische/Psychische Ursachen			
(Komorbide) Depression	*PHQ-2:* Bestanden in den letzten 2 Wochen oft Niedergeschlagenheit, Schwermut oder Hoffnungslosigkeit? Bestanden in den letzten 2 Wochen Interesse- oder Freudlosigkeit?	*→*	*Ggf. PHQ‑9* *Weiterführende psychiatrische Abklärung/Behandlung*
Suchterkrankung Negativsymptome einer Schizophrenie Hypoalertes Delir Demenzielle Entwicklung	Hinweise auf Suchtverhalten, bizarrer Wahn, Stimmenhören, Bewusstseinsstörung, kognitive Beeinträchtigung?		
**A**namnese der Medikation			
Sedierender Effekt der Begleitmedikation Erschöpfung/Müdigkeit und Schläfrigkeit als NW	*Einnahme von …* Hypnotika? Benzodiazepinen? Antidepressiva? Antiepileptika? Antipsychotika? Antihistaminika? Antihypertensiva (v. a. Alpha-2-Antagonisten)? Phasenprophylaktika?	*→*	*Evtl. Adaptierung der Medikation*
**I**nternistische Ursachen und Infektionskrankheiten			
Herz‑, Lungen‑, Leber‑, Nieren- oder Stoffwechselerkrankungen	Bestehen Auffälligkeiten im Basislabor inkl. BB, TSH, GOT, GPT, γ‑GT, Kreatinin, GFR, Eisenstatus? Klinisch relevante Herz‑, Lungen‑, Leber‑, Nieren- oder Stoffwechselerkrankungen in der Anamnese?	*→*	Weiterführende internistische Beurteilung/Behandlung PSG-Kontrolle
Autoimmun- oder Infektionskrankheiten	Hinweise auf eine Autoimmunerkrankung oder Post-COVID?		
Gründe für eine nicht effektive OSA-Therapie: Insuffiziente CPAP-Therapie REM-dominante OSA Anwendungsfehler bei CPAP Treatment emergent central sleep apnea (= zentrale Apnoen unter CPAP) Persistierende RERAs	Adhärente Anwendung der CPAP- oder Alternativtherapie (Nutzung > 4 h pro Nacht und > 70 % der Nächte in den letzten 3 Monaten)? Alter der letzten PSG > 3 Jahre? Respiratorische Auffälligkeiten in der letzten PSG?		
**N**eurologische Ursachen			
Narkolepsie Idiopathische Hypersomnie	Exploration der *red flags*: Kataplexie: Trat beim Lachen (z. B. über einen guten Witz) schon einmal eine Schwäche der Gesichtsmuskulatur, des Kopfes, des Rumpfes oder der Beine auf? Schlafparalyse: Kam es aus dem Schlaf heraus schon einmal zu einer völligen Lähmung der Gliedmaßen und Problemen, die Augen zu öffnen?	*→*	MSLT Weiterführende neurologische Abklärung/Behandlung
RLS PLMD	Besteht ein abendlicher Bewegungsdrang der Beine, verbunden mit Missempfindungen?		
Schwere neurologische Erkrankungen	Positive Anamnese hinsichtlich multiple Sklerose, Parkinson-Syndrom, Z. n. Schädel-Hirn-Trauma, neuromuskuläre Erkrankungen u. a.?		

*BIISSS* Behaviorally induced insufficient sleep syndrome, *PHQ‑2* 2-Item-Depressionsmodul PHQ‑D des Patient Health Questionnaire PHQ, *PHQ‑9* 9-Item-Depressionsmodul PHQ‑D des Patient Health Questionnaire PHQ, *RERA* respiratory effort-related arousals, *PSG* Polysomnographie, *RLS* Restless-Legs-Syndrom, *PLMD* Periodic Limb Movement Disorder, *MSLT* Multipler Schlaflatenztest

### Schlafverhalten

Zur Exploration des Schlafverhaltens ist die Ermittlung der Bettliegezeit und der tatsächlichen Schlafzeit erforderlich. Die Schlafeffizienz kann durch die anamnestische Ermittlung der Bettzeiten unter Anwendung der in Tab. 1 genannten Schlüsselfragen systematisch erfragt werden. Ihre Beantwortung kann richtungsweisend für die Diagnose z. B. eines Schlafmangels, eines insuffizienten Schlafsyndroms, einer Schlaf-Wach-Rhythmusstörung oder einer Insomnie sein.

Bei unregelmäßigen Bettliegezeiten oder einer anhaltend verkürzten Schlafdauer sollte zur genaueren Erhebung ein Schlaftagebuch und (optional) eine Aktigraphie (Aufzeichnung der Ruhe- und Aktivitätszeiten mittels Akzelerometer, für mindestens 7 oder besser 14 Tage, mit Wochentagen und Wochenendtagen) durchgeführt werden [14, 41].

#### Behaviorally induced insufficient sleep syndrome (BIISS)

Zu den häufigsten Ursachen einer erhöhten Tagesschläfrigkeit zählt das insuffiziente Schlafsyndrom (Schlafmangelsyndrom) (engl. behaviorally induced insufficient sleep syndrome, BIISS), dessen Prävalenz bei tagesschläfrigen Patienten bei etwa 7–10 % liegt [18, 27]. Hierbei handelt es sich um eine chronische, meist über Wochen oder Monate praktizierte reduzierte Schlafdauer von < 7 h pro Nacht (besonders an Wochentagen). Da die verkürzte Schlafdauer den Patienten meist zur Gewohnheit wird, wird sie häufig nicht als mögliche Ursache einer Tagesschläfrigkeit in Betracht gezogen. Die aktive Exploration ist darum für die Diagnosestellung entscheidend. Wenn nötig, sollten auch hier Schlaftagebücher und/oder eine Aktigraphie (s. oben) eingesetzt werden.

#### Schlaf-Wach-Rhythmusstörungen

Eine andauernde oder wiederkehrende Störung des Schlaf-Wach-Rhythmus entsteht durch eine Inkongruenz zwischen endogenem zirkadianen Rhythmus (innere Uhr) und dem Schlaf-Wach-Plan, der durch die individuelle physische Umgebung oder soziale und berufliche Umstände gefordert wird.

Neben der bekanntesten Kurzform, dem Jetlag, zählt zu dieser Gruppe unter anderem das Schichtarbeitersyndrom [41], das ein Risiko für eine exzessiv gesteigerte Tagesschläfrigkeit bei gleichzeitig bestehenden insomnischen Beschwerden darstellt.

### Depressionsscreening

Etwa 14 % der an einer Depression erkrankten Personen beklagen rein hypersomnische Symptome (ohne Insomnie) [12], die sich als exzessiv gesteigerte Tagesschläfrigkeit bemerkbar machen können. Ferner ist bekannt, dass etwa 35 % aller OSA-Patienten auch an einer klinisch signifikanten Depression leiden [9]. Es überrascht darum nicht, dass klinische Studien den Verdacht bestätigen konnten, dass Patienten mit pathologisch gesteigerter Tagesschläfrigkeit auch überdurchschnittlich häufig hohe Depressionsscores aufweisen [19, 42].

Für das Screening einer Depression eignet sich besonders der Patient Health Questionnaire PHQ‑2, der u. a. auch in der Leitlinie „Müdigkeit“ der Deutschen Gesellschaft für Allgemeinmedizin und Familienmedizin (DEGAM) empfohlen wird. Er besteht aus 2 Fragen, die in Tab. 1 als Schlüsselfragen wiedergegeben sind. Wenn beide Fragen des PHQ‑2 verneint werden, kann eine depressive Episode (DSM: major depression) mit hoher Wahrscheinlichkeit ausgeschlossen werden (Sensitivität: 87 %) [22, 43]. Zur näheren Differenzierung des Schweregrades einer depressiven Episode bietet sich etwa der als Screeninginstrument im nicht-psychiatrischen Fachbereich entwickelte PHQ‑9 (9-Item-Depressionsmodul PHQ‑D des Patient Health Questionnaire PHQ) an [20, 32].

### Fatigue bei Autoimmun- und Infektionskrankheiten

Eine ausgeprägte und lähmende Müdigkeit oder Erschöpfung (Fatigue) ist die häufigste Beschwerde, die Menschen mit Autoimmunerkrankungen wie etwa systemischem Lupus erythematodes, Typ-1-Diabetes, Zöliakie, chronischer Hepatitis, myalgischer Encephalomyelitis/Chronic Fatigue Syndrome oder rheumatoider Arthritis beklagen [47].

In der COVID-19-Pandemie wurde die Fatigue-Symptomatik zum Massenphänomen: Müdigkeit und Erschöpfung sind nicht nur typische Symptome dieser Infektionserkrankung, sondern verbleiben auch häufig als Teil der Residualsymptomatik (Post-COVID-Syndrom). Einer rezenten Metaanalyse zufolge, leidet etwa ein Drittel der Patienten, die eine COVID-Infektion überstanden haben, an Fatigue-Symptomen [4].

Ausmaß und Tragweite dieses Leidens sind zum jetzigen Zeitpunkt nicht abschätzbar [44].

### Zentrale Störungen mit Hypersomnolenz

Schlüsselsymptom der Hypersomnolenzerkrankungen ist die exzessiv gesteigerte Tagesschläfrigkeit, die in der differentialdiagnostischen Abklärung bei Persistenz von Tagesschläfrigkeit bei gut behandeltem OSA erwogen werden muss. Zu diesen Erkrankungen gehören die Narkolepsie Typ 1 und Typ 2 und die idiopathische Hypersomnie.

Bei der Narkolepsie treten neben der exzessiven Tagesschläfrigkeit häufig weitere REM-Schlaf-assoziierte Symptome wie Kataplexien, Schlafparalysen, automatisches Handeln und hypnagoge und/oder hypnopompe Halluzinationen auf. Von besonders hohem diagnostischen Wert ist die Exploration des Vorliegens von Kataplexien (pathognomonisch für Narkolepsie Typ 1), bei welchen es zu einem affektgetriggerten (meist in positiven emotionalen Situationen) Verlust der Muskelspannung, meist beginnend in der Nackenmuskulatur oder dem Gefühl eines Tonusverlustes in den Beinen bei vollständig erhaltenem Bewusstsein kommt. Hierzu können die beiden Schlüsselfragen in Tab. 1 dienen.

Bei Anamnese einer exzessiv gesteigerten Tagesschläfrigkeit sollte bei Vorliegen von weiteren narkolepsietypischen Symptomen eine weiterführende neurologische und schlafmedizinische Untersuchung mittels MSLT zur Beurteilung der Einschlaflatenz und des frühen Auftretens von REM-Schlaf durchgeführt werden. Ergänzend können zudem eine HLA-Typisierung (HLA DQB1*0602) und eine Hypocretinbestimmung aus dem Liquor zur Diagnosesicherung einer Narkolepsie Typ 1 erfolgen [17]. Zudem sollte bei allen Formen der zentralen Störungen mit Hypersomnolenz eine Aktigraphie erfolgen.

### Restless-Legs-Syndrom (RLS)

Das RLS ist bei bis zu 80 % der Betroffenen mit periodischen Beinbewegungen assoziiert. Diese führen nicht immer zu einer Nachtschlaffragmentierung und sind somit nicht grundsätzlich für eine gesteigerte Tagesschläfrigkeit verantwortlich, allerdings kann durch ein RLS eine Insomnie verursacht oder aufrechterhalten werden, welche eine Tagesschläfrigkeit hervorrufen oder verstärken kann [15].

Ein Zusammenhang einer Tagesschläfrigkeit mit isolierten, periodischen Beinbewegungen während des Schlafes (engl. periodic leg movements [PLM]) ohne das Vorliegen eines RLS ist gegenwärtig Gegenstand kritischer Diskussion [7, 13].

## Medikamentöse Behandlung der rETS

Nur wenn eine suffiziente CPAP-Adhärenz oder adäquate Alternativtherapie und der Ausschluss möglicher Differentialdiagnosen (SPAIN-Checkliste) erfolgt ist, sollte die Diagnose einer rETS gestellt werden. Das Vorliegen einer rETS erlaubt die Indikation einer symptomatischen Therapie. In Europa stehen zur Behandlung der rETS derzeit grundsätzlich verschiedene, die Wachheit fördernde Medikamente zur Verfügung. Zu diesen gehören Solriamfetol, Pitolisant, Modafinil, Methylphenidat und Amphetamine. Die gegenwärtig einzig zugelassene Substanz zur Behandlung der rETS ist Solriamfetol. Für Pitolisant besteht eine Zulassungsempfehlung des CHMP (Ausschuss für Humanarzneimittel der Europäischen Arzneimittelbehörde EMA – European Medical Agency). Die Zulassung für Modafinil zur Behandlung der Tagesschläfrigkeit bei OSA wurde aufgrund der unzureichenden Studienlage und des ungünstigen Nutzen-Risiko-Profils von der EMA im Jahr 2010 wieder zurückgenommen.

Tab. 2 zeigt die o. g. Substanzen, die einen möglichen Effekt auf die Verbesserung einer rETS haben können.

**Table 2:** 

	Solriamfetol	Pitolisant	Modafinil
**–**	Selektiver Noradrenalin-Dopamin-Wiederaufnahmehemmer	Antagonist und inverser Agonist am Histamin-H3-Rezeptor und Modulator von Dopamin, Acetylcholin und Noradrenalin	Selektiver α1-Agonist und Modulator von Dopamin, Serotonin oder Noradrenalin
*Zulassung*	Ja	Zulassungsempfehlung der CHMP der EMA	Nein
*Halbwertszeit*	6–8 h	10–12 h	3–4 h
*Dosierung*	Initial 37,5 mg morgens, je nach Ansprechen Dosisverdopplung alle 3 Tage bis zu einer max. Tagesdosis von 150 mg	1. Woche: 2 × 4,5 mg (morgens und mittags), je nach Ansprechen wöchentliche Dosisverdopplung bis zu einer max. Tagesdosis von 2 × 18 mg	1. Woche: 100 mg, 2. Woche: 200 mg tgl. (morgens oder morgens und mittags)
*Gegenanzeigen*	Myokardinfarkt innerhalb des vergangenen Jahres, instabile Angina pectoris, unkontrollierte Hypertonie, schwerwiegende Arrhythmien oder andere schwerwiegende Herzprobleme Nicht gleichzeitig mit oder < 14 Tage nach Beendigung der Behandlung mit Monoaminoxidasehemmern (MAO-Hemmern) Dosisanpassung bei Niereninsuffizienz Kontrazeption erforderlich Keine Anwendung während Schwangerschaft und Stillzeit	Schwere Leberfunktionsstörung (Child-Pugh C) Dosisanpassung bei Niereninsuffizienz Nichthormonelle Kontrazeption erforderlich Keine Anwendung während Schwangerschaft und Stillzeit	(Mittel)schwere Hypertonie Herzrhythmusstörungen Nichthormonelle Kontrazeption erforderlich Keine Anwendung während Schwangerschaft und Stillzeit
*Häufigste Nebenwirkungen*	Kopfschmerzen, Übelkeit und Appetitverlust	Kopfschmerzen, Übelkeit, Schwindel, Insomnie, Appetitverlust, Ängste und Rhinopharyngitis	Kopfschmerzen, Hautreaktionen, Schwindel, Bluthochdruck, Depression, Suizidgedanken, Psychosen
*Beobachtete Wirkung/Vorteile*	Durchschnittliche Senkung des ESS um 7,7 Punkte (nach 12 Wochen) Steigerung der Einschlaflatenz um +11 min im MWT Keine Rebound-Hypersomnolenz oder Entzugserscheinungen nach Absetzen Langzeitsicherheit bestätigt [23, 37, 39]	Durchschnittliche Senkung des ESS um 2,6 Punkte (20 mg in 3 Tagen) bzw. um 5,9 Punkte (40 mg in 12 Wochen). Kein Effekt auf Einschlaflatenz im MWT [5, 30, 36]	Durchschnittliche Senkung des ESS um 2,2 Punkte. Steigerung der Einschlaflatenz um +15 min im MWT [3, 28]

*EMA* Europäischen Arzneimittelbehörde, *CHMP* Ausschuss für Humanarzneimittel der EMA, *ESS* Epworth Sleepiness Scale, *MWT* Multipler Wachbleibetest

Die individuelle Therapieentscheidung der symptomatischen Behandlung der residualen Tagesschläfrigkeit mit Substanzen, die eine Tageswachheit fördern, sollte immer den aktuellen Empfehlungen der zur Verfügung stehenden Präparate, den individuellen Bedürfnissen und den vorliegenden Begleiterkrankungen angepasst werden. Sie sollte außerdem im Behandlungsverlauf regelmäßig hinterfragt und bei Bedarf adaptiert werden.

## Fazit für die Praxis

- Die exzessive Tagesschläfrigkeit (rETS), die bei 6–10 % der Patienten mit suffizient behandelter OSA zu finden ist, sollte als behandlungswürdiges Symptom wahrgenommen werden.
- Sie stellt eine differentialdiagnostische Herausforderung dar und erfordert im Management der OSA eine interdisziplinäre Herangehensweise (SPAIN-Checkliste).
- Für die Behandlung der rETS stehen effektive Therapeutika zur Verfügung, die für die Patienten individuell nutzbar gemacht werden sollten.
